# Recurrence, Reactivation, or Inflammatory Rebound of SARS-CoV-2 Infection With Acute Vestibular Symptoms: A Case Report and Revision of Literature

**DOI:** 10.3389/fnhum.2021.666468

**Published:** 2021-08-11

**Authors:** Salvatore Zaffina, Paola Lanteri, Francesco Gilardi, Sergio Garbarino, Annapaola Santoro, Maria Rosaria Vinci, Rita Carsetti, Alessandro Scorpecci, Massimiliano Raponi, Nicola Magnavita, Vincenzo Camisa

**Affiliations:** ^1^Health Directorate, Occupational Medicine Unit, Bambino Gesù Children's Hospital, IRCCS, Rome, Italy; ^2^Post-graduate School of Occupational Health, Section of Occupational Medicine and Labor Law, Università Cattolica del Sacro Cuore, Rome, Italy; ^3^Department of Diagnostics and Applied Technology, Neurophysiopathology Centre, Fondazione IRCCS, Istituto Neurologico “Carlo Besta”, Milan, Italy; ^4^Italian Ministry of Health, Rome, Italy; ^5^Department of Neuroscience, Rehabilitation, Ophthalmology, Genetics and Maternal/Child Sciences, University of Genoa, Genoa, Italy; ^6^Department of Laboratories, Unit of Diagnostic Immunology and Immunology Research Area, Unit of B-Cell Pathophysiology, Bambino Gesù Children's Hospital, IRCCS, Rome, Italy; ^7^Audiology and Otosurgery Unit, Bambino Gesù Children's Hospital, IRCCS, Rome, Italy; ^8^Health Directorate, Bambino Gesù Children's Hospital, IRCCS, Rome, Italy; ^9^Department of Woman, Child and Public Health, Fondazione Policlinico Universitario A. Gemelli IRCCS, Rome, Italy

**Keywords:** recurrence, occupational medicine, neuroCOVID, neurologic symptoms, vestibular syndrome, healthcare workers, case report

## Abstract

A case of recurrent coronavirus disease 2019 (COVID-19) with neurovestibular symptoms was reported. In March 2020, a physician working in an Italian pediatric hospital had flu-like symptoms with anosmia and dysgeusia, and following a reverse transcription PCR (RT/PCR) test with a nasopharyngeal swab tested positive for SARS-CoV-2. After home quarantine, 21 days from the beginning of the symptoms, the patient tested negative in two subsequent swabs and was declared healed and readmitted to work. Serological testing showed a low level of immunoglobulin G (IgG) antibody title and absence of immunoglobulin M (IgM). However, 2 weeks later, before resuming work, the patient complained of acute vestibular syndrome, and the RT/PCR test with mucosal swab turned positive. On the basis of the literature examined and reviewed for recurrence cases and vestibular symptoms during COVID-19, to our knowledge this case is the first case of recurrence with vestibular impairment as a neurological symptom, and we defined it as probably a viral reactivation. The PCR retest positivity cannot differentiate re-infectivity, relapse, and dead-viral RNA detection. Serological antibody testing and viral genome sequencing could be always performed in recurrence cases.

## Introduction

In China, in December 2019, the epidemic caused by severe acute respiratory syndrome-coronavirus-2 (SARS-CoV-2) rapidly diffused all over the world leading to a pandemic. While the coronavirus disease 2019 (COVID-19) typically presents as a self-limiting respiratory disease, and in hospitalized patients, the clinical picture is dominated by respiratory distress (Del Sole et al., 2020), progression to severe illness with multiorgan involvement, including the blood vessels, heart, gut, kidneys, testicles, and brain has been reported (Asadi-Pooya and Simani, 2020; Chen X. et al., 2020; Ibrahim, 2020; Leonardi et al., 2020; Nepal et al., 2020). Patients suffering from COVID-19 can develop acute or long-term neurological sequelae (Ellul et al., 2020). The prevalence of neuro-COVID varies considerably between individual studies ranging from 4.1% (Xiong et al., 2020) to 57.4% (Romero-Sánchez et al., 2020) and even 84% in COVID-19 with acute respiratory distress syndrome (Helms et al., 2020). The onset of nervous system damage can be asynchronous with systemic manifestations and the typically salient severe respiratory disease (Vavougios, 2020).

In our pediatric hospital, between March and May 2020, there were 25 cases of COVID-19 among healthcare workers (HCWs), equal to 1.1% of the total staff. Two of them had at least one symptom, namely, headache, anosmia, and dysgeusia or all the three symptoms at the same time. These neurological symptoms indicate that the virus, like other respiratory viruses (Bohmwald et al., 2018), enters the central nervous system (CNS) through the olfactory bulb causing inflammation. Furthermore, SARS-CoV-2 viruses can spread from the mechano- and chemo-receptors in the lungs and lower respiratory airways to the medullary cardiorespiratory center *via* a synapse-connected route (Li Y. C. et al., 2020).

In this study, we report a case of recurrent SARS-CoV-2 infection with neurovestibular involvement, review literature cases with vestibular involvement, and discuss the neurotropism of this virus based on literature data.

### Case Description

**Figure 1:**
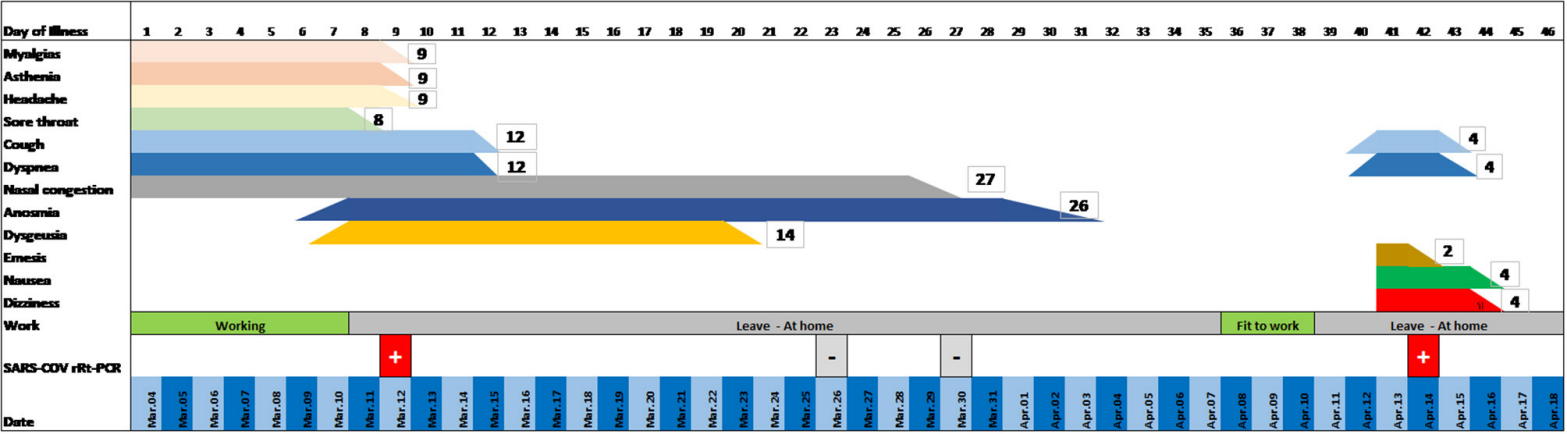
Clinical evolution of the patient: timeline.

A 48-year-old female physician in a pediatric hospital presented cough, slight dyspnea, severe myalgia, asthenia, and headache on March, 2020, followed by anosmia and dysgeusia over the following few days (Figure 1) without fever. When symptoms appeared the worker self-isolated at home. The allergic rhinitis the patient suffers from led to a short delay in diagnosis; however, a swab carried out 8 days from the beginning of the symptoms tested positive for SARS-CoV-2 (Allplex™2019-nCoV Assay). In subsequent days, the patient felt better and became asymptomatic. On days 23 and 27 the patient was retested and was negative both times. On day 37, serology was performed by ELISA and a low level of immunoglobulin G (IgG) against SARS-CoV-2 was detected. Immunoglobulin M (IgM) search was negative. She was feeling well and was declared fit to work by the occupational physician of the hospital. However, before returning to work, on day 39 the patient woke up experiencing intensive dizziness, described as subjective vertigo, associated with vomiting and bilateral aural fullness (Figure 1). The dizziness quickly got worse as the patient lied supine on the right side. She was transported by ambulance to the emergency room of a hospital where dizzying syndrome was diagnosed and treated with metoclopramide. Physical examination revealed normal vital signs, while the patient was breathing ambient air. Some relevant auxiliary examinations such as blood routine, coagulation function, liver and renal function, electrolytes, and inflammation indicators were completed, and the results were normal. After 3 h and the improvement of the symptoms, she was discharged home, cared by health public service, and treated with betahistine dihydrochloride for 1 week. The day after, on day 40, a nasal swab was obtained, which tested positive.

In the following days, the dizziness disappeared and only a mild postural instability persisted, whereas aural fullness was unchanged. The only pathology that she suffered from was allergic rhinitis. She had never suffered from dizziness in the past.

An ear, nose, and throat (ENT) visit took place on day 44. Otoscopy was negative. Romberg, Unterberger (March-in-place), and finger-to-nose tests were negative. Neither bedside vestibular examination with Frenzel glasses showed spontaneous nystagmus, nor was nystagmus evoked by supine position, Dix-Hallpike maneuver, lateral head rotation, and Rose position. The head-shaking test was negative. The only relevant finding of the vestibular examination was the patient complaining about slight dizziness during the Dix-Hallpike maneuver on the right side (supine position with the head hyperextended and tilted to the right side). The patient did not develop fever both in the first and in the second phase of the symptoms related to COVID-19. Despite fever being one of the most common symptoms of COVID-19, it may be absent in some individuals. Finally, the patient was completely healed without any deficit and was able to return to work.

## Discussion

The case reported presents numerous points of interest. First, the recurring nature of the symptoms must be emphasized along with the neurological origin of the symptoms themselves. Second, the neurotropism and neuroinvasive potential of the virus into recurrence mode should be considered. Finally, the observation that the presence of anti-spike IgG has been followed by a positive PCR test; this feature is rather infrequent, having been found in 0.13 per 10,000 days at risk in HCWs, whereas the probability of having a positive PCR test in the anti-spike–seronegative HCWs is significantly higher (1.09 per 10,000 days at risk) (Lumley et al., 2020).

The presence of at least two consecutive negative RT-PCR tests in respiratory samples (with samples taken at least 24 h apart) and the appearance of specific IgG at serological test, according to the discharge criteria of the European Centre for Disease Prevention and Control (Yahav et al., 2021), permits to classify the case exposed as recurrent COVID-19 with multiple district neurological symptoms (vestibular symptoms, previously anosmia and dysgeusia). Indeed reinfection should be considered during the first 90 days if clinical symptoms of the first episode resolve and two PCR tests were negative before the new episode (Yahav et al., 2021).

Currently, there are numerous reports that a number of patients tested positive again after two consecutive negative PCR tests or after clinical recovery (Table 1) (Falahi and Kenarkoohi, 2020). Nasopharyngeal swabs tend to have a higher sensitivity than the other samples and are the most common method for diagnosis of COVID-19 recurrence, regardless of the clinical disease manifestation. With the progression of SARS-CoV-2 infection, the virus could migrate from the upper respiratory tract to the lower respiratory tract and lungs, resulting in insufficient viral load in the upper respiratory tract, which may explain the negative result of the nasopharyngeal swab test. The detection rate and sensitivity have been improved by multisite specimen collection and serological assays (Table 1). The prevalence of disease recurrence among COVID-19 recovered patients was approximately 14.8%, ranging from 7.35 to 21.4% (Azam et al., 2020; Hoang, 2020). The interval duration from the last negative PCR tests to recurrent positive results ranged from 1 to 123 days (min average 16.16 ± 20.93 ds, max average 25.39 ± 23.00 ds) for 1,038 cases in a selected population of 13,565 patients (Table 1). The case described experienced at the onset two of the three typical symptoms in the first phase of infection, namely, cough (68%) and shortness of breath (66%) without fever (69%). About 92% of the patients experienced at least one of these, less frequently in women (66, 64, 66 respectively; 90% at least one of these; vs. men 70, 67, 71; 93%, each *p* < 0.001) (ISARIC Clinical Characterisation Group, 2021). She presented symptoms of recurrence 17 days from the last negative PCR tests, in accordance with the literature. True reinfection has criteria that must be considered, including isolation of the complete genome of the virus (and not just genomic fragments) in the second episode (Falahi and Kenarkoohi, 2020), but we did not have this data. In our patient, we could suspect a viral reactivation due to low level of IgG against SARS-CoV-2 detected, even without lymphopenia. It is also possible that the immune responses can suppress, but not completely eradicate, SARS-CoV-2, which may have led to the false-negative results due to lower viral loads (Chen J. et al., 2020). Once the virus starts replicating again, the RT-PCR results reverted to positive and expressed as a new neuroinvasion in the vestibular system. NeuroCovid is now well-known (Whittaker et al., 2020), but recurrence of positive RT-PCR with neurological symptoms is very rare and no further instances of vestibular symptoms are described as recurrence (Table 1). To our knowledge, this is the first case of vestibular symptoms as recurrence of positive RT-PCR SARS-CoV-2. This case shows the neuroinvasive potentials of SARS-CoV-2 and the possibility of disease reactivation after clinical and analytic recovery. SARS-CoV-2 might be latent in some neurons to hide from immune surveillance (Brandt and Dieterich, 2017; Zhang M. et al., 2020). For reasons unclear, after an apparent remission the virus reactivated and was again identifiable in the respiratory tract. At the same time, the multiplication in the nervous system caused recurrence with intense neurological disturbance, clinically highlighted by dizziness associated with slight dyspnea.

**Table 1:** Recurrent cases reported in literature.

**Reference**	**Patients (n°)**	**Days from recurrence of COVID-19**	**Test for recurrence of SARS-CoV-2 positive**	**Kit type (sensitivity and specificity)**	**Clinical symptoms**	**Recurrence with neurological signs**
Abdullah et al., 2020	27/138 pt	11 days	Nasopharyngeal and oropharynx swabs for RT-PCR	No specified	6 pts mild symptoms, 21 pts no clinical symptoms	
Alonso et al., 2020	26-year-old man	1 month later	Nasopharyngeal and throat swabs for RT-PCR	Allplex™2019-nCoV Assay [sensitivity (95% CI) 98.2 (90.3–100.0) % specificity (95% CI) 100.0 (94.9–100.0)]	A more vigorous COVID-19 recurrence	
An et al., 2020	38/242 pts	5–7 days	Digestive (anal swab) and respiratory RT-PCR tests for the S gene and for ORF genes. *C_t_* ≤ 37.0 Next-generation sequencing of samples Specific total antibody IgG, IgA, and IgM	QIAamp RNA Viral Kit (hyper-sensitive kit compares to commercial kit)	Fewer respiratory and digestive tract symptoms	
Bentivegna et al., 2020	1 pt	23 days	Nasopharyngeal swab RT-PCR IgM seroconversion	No specified	Asymptomatic	
Bongiovanni et al., 2020	125/1,146 pts	Mean 19.9 (3–43) days	Nasopharyngeal swabs RT-PCR	No specified	Asymptomatic (96, 76.8%), general sign (25, 20.0%), and respiratory failure (4, 3.2%)	
Cao et al., 2020	8/108 pts	6–28 days	Deep nasal cavity swab samples or throat swab samples RT-PCR	No specified	Asymptomatic	
Cento et al., 2020	264/2,521 pts after one negative results by RT-PCR assay	20–30 days	Nasopharyngeal swabs RT-PCR *C_*t*_*s-values ≥ 24	No specified	No clinical symptoms	
Chae et al., 2020	1 pt	6 days	Naso- and oropharyngeal swab RT-PCR	No specified	Ground-glass opacities in the right upper lobe	
Chen D. et al., 2020	46-year-old woman	2 days after 2 negative tests	Oropharyngeal swab test RT-PCR	No specified	Respiratory symptoms had already improved	
Chen J. et al., 2020	81/1,067 pts	7–10 days	Throat-swabs RT-PCR tests	No specified	84.0% (68) mild, 14.8% (12) severe, and 1.2% (1) critical of the cases with pulmonary, liver, kidney, and myocardial damage	
Chen M. et al., 2020	6/11 pts	6–27 days	Oropharyngeal swab RT-PCR	No specified	Mild to moderate	
Chen S. L. et al., 2020	189/1,282 pts	28 days	Nasopharyngeal and anal swabs specimens RT-PCR ORF1ab and N genes	Catalog no. DA0931; DaAn Gene, Guangzhou, China (unavailable)	Cough (15.87%), Diarrhea (0.53%), Dyspnea (3.70%)	Fatigue (1.06%), Myalgia (1.06%),
Chen Y. et al., 2020	4 pts	3 days after discharge	Nasopharyngeal, oropharyngeal, and anal swabs RT-PCR test	Kits from different manufacturers. No specified	No clinical symptoms	
Crouwel et al., 2020	28-year-old female	50 days	Nasopharyngeal swabs for the RT-PCR test	No specified	Diarrhea, nausea, coughing, sneezing	Headache, myalgia, anosmia, and dysgeusia
Deng et al., 2020	61 pts	Ranged from 3 to 35 days (median, 10 days)	Nasal and pharyngeal swab specimens, stool and sputum specimens RT-PCR	No specified	Mild 38 (62.3%) General 20 (32.8%) Severe 3 (4.9%)	Headache 5 pts (8.2%)
Dou C. et al., 2020	1 pt	15 days	Oropharyngeal swab RT-PCR	No specified	Asymptomatic	
Dou P. et al., 2020	2 pts	17 days	Throat and anus swab RT-PCR	No specified	Asymptomatic	
Du et al., 2020	3/126 pts	10–18 days	Nasopharynx and oropharynx swab RT-PCR targeting the ORF1ab gene and N gene *C_*t*_* ≤ 37	Bio-Germ, Shanghai, China (sensitivity 96.15% specificity 100.0%)	Asymptomatic	
Duggan et al., 2021	1 pt	10 days	Nasopharyngeal swabs for the RT-PCR test	No specified	Critical	
Fu et al., 2020	3	1–5 days	Nasopharyngeal swab RT-PCR; IgM and IgG antibodies	No specified	Asymptomatic	
Gao et al., 2020	70-year-old male patient	15 days	Nasopharyngeal, blood, and rectal swab RT-PCR ORF1ab and N genes	No specified	No symptoms	
Geling et al., 2020	24-year-old male	7 days after discharge	Sputum specimen RT-PCR for ORF1ab and the N gene	No specified	No clinical symptoms	
Gidari et al., 2021	9 pts	14–50 days	Respiratory samples RT-PCR for E gene, gene N *C_*t*_* ≤ 40	Allplex™ 2019-nCoV Assay [sensitivity (95% CI) 98.2 (90.3–100.0) % specificity (95% CI) 100.0 (94.9–100.0)]	No clinical symptoms, retrosternal sense of weight	Headache, arthro-myalgias, asthenia, and insomnia
Gousseff et al., 2020	11 pts	21–49 days after a symptom (quarantine)	Naso-pharyngeal swabs RT-PCR	No specified	Median duration of symptoms was 18 days for the first episode and 10 days for the second one	
Guo et al., 2020	27-year-old man	3 days after discharge	Throat swab specimens A fluorescent immunochromatography detection kit specific to the IgM and IgG antibodies against SARS-CoV-2	Zhongshan Chuangyi Biochemical Engineering Co. (unavailable)	No clinical symptoms	
Habibzadeh et al., 2020	9/13 pts	15–48 days	Nasopharyngeal swabs RT-PCR tested the E and RdRP genes	Invitrogen ChargeSwitch Total RNA Cell Kit, Invitrogen Co. (unavailable)	No clinical symptoms	
Hao et al., 2020	24/104 pts	Quarantine	Respiratory specimens (nasal/throat swab or sputum) RT-PCR tests of	No specified	No clinical symptoms	
He et al., 2020	1 pt	8 days	Throat swab samples RT-PCR	No specified	Dry cough, arthralgia	Headache
Hu R. et al., 2020	11/69 pts	14 days (range, 9–17 days).	Nasopharyngeal swabs RT-PCR	No specified	No clinical symptoms	
Huang et al., 2020	69/414 pts	Median 19 days, range 6–52 days	Nasopharyngeal and anal swabs qRT-PCR ORF 1ab and N genes. *C_*t*_* ≤ 37 Antibody Chemiluminescent microparticle immunoassay kit IgM and IgG in plasma.	QIAamp RNAViralKit (hyper-sensitive kit compares to commercial kit code. GZ-D2RM25, Shanghai GeneoD)	Respiratory symptoms including cough and increased sputum	
Jiang et al., 2020	6/35 pts	9–10 days	Throat swabs or sputum samples for RT-PCR	No specified	1 expectoration, nausea, 1 cough, 4 asymptomatic	1 fatigue, sore muscles
Kang, 2020; KCDA, 2020	292/8,922 pts	1–37 days	Nasopharyngeal swabs RT-PCR	No specified	Asymptomatic to minor symptoms	
Landi et al., 2020	6/29 pts	13–24 days	Nasopharyngeal swabs RT-PCR	No specified	Asymptomatic or mild	
Lan et al., 2020	4 pts	5–13 days	Throat swabs RT-PCR	BioGerm (sensitivity 96.15% specificity 100.0%)	Asymptomatic	
Li C. et al., 2020	15/85 pts	9–30 days	Nasopharyngeal swabs RT-PCR	No specified	Two patients (13.3%) had cough, one (6.6%) had dyspnea	
Li J. et al., 2020	50-year-old man	Days 34 and 38 during quarantine	Nasopharyngeal swabs RT-PCR	No specified	No clinical symptoms	
Li Y. et al., 2020	6/13 pts	6–14 days	Respiratory swabs qRT-PCR for RdRP, E, and N gene	Liferiver detection kit (sensitivity 90% specificity 100.0%)	Asymptomatic	
Ling et al., 2020	11/66	6–11 days	Oropharyngeal swab, stool, urine, and serum RT-PCR	Biosystems 251658240 7500 Real-Time PCR Systems (sensitivity 85.3% specificity 100.0%)	Asymptomatic	
Liu B. et al., 2020	8/47 pts	From 8 to 39 days after viral shedding	Anal and throat swab samples RT-PCR Antibodies against the spike glycoprotein (S); the receptor-binding domain (RBD); conserved heptad repeats (HR1–HR2) in the S2 domain; and the N, membrane (M), and E proteins.	Kit from a different manufacturer (no specified)	No clinical symptoms	
Liu et al., 2020a	9/51 pts	7–14 days	Oropharyngeal-swab RT-PCR	BioGerm (sensitivity 96.15% specificity 100.0%)	Asymptomatic (6, 66.7%), mild (3, 33.3%)	
Liu F. et al., 2020	35-years old man	Positive during quarantine and returned positive after second quarantine (three hospitalizations)	Nasopharyngeal swabs RT-PCR E gene, RdRP gene, and N gene *C_*t*_* ≤ 43	Liferiver detection kit (sensitivity 90% specificity 100.0%)	Mild clinical symptoms deteriorations	
Liu J. et al., 2020	15/62 pts	14 days	Respiratory tract samples RT-PCR spike receptor-binding domain (S-RBD) and N spike protein as antigens. ORF1ab, NP genes fragments, Ct ≤ 38 COVID-19 IgG or IgM antibody	BioGerm (sensitivity 96.15% specificity 100.0%)	Mild cases 5 (33.3%) Common cases 9 (60.0%) Severe cases 1 (6.6%)	
Liu T. et al., 2020	11/150 pts	38 days, range 35–44 days	Throat swabs RT-PCR and serum IgM/IgG rapid test	BioGerm (sensitivity 96.15% specificity 100.0%)	No clinical symptoms reported	
Loconsole et al., 2020	48-year-old man	30 days after 2 negative tests	Nasopharyngeal swab RT-PCR 2 targeting E-gene, RdRP-gene and N-gene	No specified	New symptoms, i.e., dyspnea and chest pain.	
Lu et al., 2020	87/619 pts	2–19 days	Nasopharyngeal swabs, throat swabs and anal swabs RT-PCR and multiplex PCR sequencing including targeting the ORF1ab, N, RdRp, E. Microneutralization antibody assays for SARS-CoV-2	**Three kits** DAAN GENE (unavailable) BioGerm (sensitivity 96.15% specificity 100.0%) Liferiver detection kit (sensitivity 90% specificity 100.0%)	Asymptomatic (77, 88.5%), mild (10, 11.5%)	
Luciani et al., 2020	69-year-old man	41 days	Nose-pharyngeal swab RT-PCR	No specified	Fever, dyspnea, anemia	
Mardani et al., 2020	64-year-old woman	21 days	Nasopharyngeal swabs RT-PCR	QiaSymphony; Qiagen, Hilden, Germany (hyper-sensitive kit compares to commercial kit)	Consciousness suddenly decreased, associated with respiratory distress	Meningoencephalitis
Mei et al., 2020	23/651 pts	4–38 days	Nasopharyngeal and oropharyngeal swabs qRT-PCR immunochromatographic strip assay for anti-SARS-CoV-2 viral immunoglobulins	No specified	15 (65%) were asymptomatic, 8 presented mild to moderate symptoms	
Peng et al., 2020)	7 pts	During quarantine	Throat or anal swab on qRT-PCR	No specified	Milder symptoms	
Qiao et al., 2020	1/15 pts	16 days	Throat swabs RT-PCR	No specified	Mild (itchy throat)	
Ravioli et al., 2020	2 pts	14–21 days	Nasopharyngeal swab RT-PCR	No specified	Moderate (1, 50.0%) and death (1, 50.0%)	
Salcin and Fontem, 2020	62 year old female	120 days	Nasopharyngeal swabs RT-PCR	No specified	Acute Respiratory Distress Syndrome	
Sen et al., 2020	5 pts	5–43 days	Nasopharyngeal swabs RT-PCR	No specified	1 pt asymptomatic/ 4 pts acute febrile illness	
Sharma et al., 2020	57-year-old man	48 days	Nasopharyngeal swabs RT-PCR RdRp gene and E gene. Ct ≤ 30 Rapid COVID-19 IgM and IgG	Cephpeid Xpert® Xpress (unavailable)	Fever, and a productive cough	Myalgia, headache
Fernandes Valente Takeda et al., 2020	6 pts health professionals	ranged from 53 to 70 days (median, 56.5 days)	Naso and/or oropharyngeal swab samples RT-PCR	No specified	Symptomatic second episode	2 pts anosmia
Tian et al., 2020	20/147 pts	17.25 days, ranging 7–47 days after discharge	RT-PCR ORF1ab gene and N gene	DAAN GENE, Guangzhou, China (unavailable)	No clinical symptoms	
To et al., 2020	1 pt	123 days	Respiratory specimens RT-PCR whole genome sequencing	LightMix® E-gene kit (highly sensitive, specificity 100%).	Asymptomatic	
Wang H. et al., 2020	1 pt	15 days	Sputum, nasopharyngeal swabs RT-PCR	No specified	Mild	
Wang X. et al., 2020	8/131 pts	7–30 days	Nose and throat RT-PCR ORF1b and N	No specified	2 pts fever, 6 pts no clinical symptoms	
Wong et al., 2020	21/106 pts	13–16 days	Nasopharyngeal swab RT-PCR Orf1ab and N Ct <40	BGI Genomics [sensitivity 88.2%e (78.1–94.8), specificity 100% (95.8–100)]	1 a mild cough, 20 asymptomatic	
Wu F. et al., 2020	1 pt	6 days	Throat swab RT-PCR	No specified	Mild	
Wu J. et al., 2020	10/60 pts	In-home 2-week quarantine	Nasopharyngeal and anal swab samples RT-PCR	No specified	2 occasionally cought, 8 no clinical symptoms	
Xiao A. T. et al., 2020	15 of 70 patients	45 days after symptoms onset	Throat swab samples or deep nasal cavity swab samples RT-PCR	Shanghai Huirui Biotechnology Co., Ltd. (unavailable)	No symptoms	
Xiao Y. et al., 2020	40/116 pts	During quarantine	Nasopharyngeal swab RT-PCR	Shanghai Huirui Biotechnology Co. (unavailable)	No symptoms	
Xie et al., 2020	22/161 pts	1–14 days	Throat swabs and anal swabs RT-PCR	No specified	No data	
Xing et al., 2020	2/62 pts	6–14 days	Throat swab samples RT-PCR for ORF1ab and N. *C_*t*_* ≤ 37	BioGerm (sensitivity 96.15% specificity 100.0%)	Asymptomatic	
Yang et al., 2020	93/479 pts	7-90 days	RT-qPCR *C_*t*_* ≤ 40 IgM, IgG, and total antibody	Zhongshan Daan Biotech. (sensitivity and specificity 100%)	67 (72%) no symptoms, while 26 (28%) mild symptoms, including slight cough (18/93 [19%]) and chest tightness (3/93 [3%]).	
Ye et al., 2020	5/55 pts	30 days	Throat swab samples qRT-PCR	No specified	Fever	
Yoo et al., 2020	1 pt	14 days	Upper airway (nasopharyngeal swab), lower airway (sputum), urine, stool, saliva, and serum. qRT-PCR RdRP, N genes, and E gene *C_*t*_*-values <35	Allplex™2019-nCoV Assay (Seegene Inc., Seoul, Korea) [sensitivity (95% CI) 98.2 (90.3–100.0) % specificity (95% CI) 100.0 (94.9–100.0)]	Mild	
Yuan B. et al., 2020	20/182 recovered patients	During a 14-day medical isolation	Blood, nasopharyngeal swabs, and anal swabs RT-PCR Antibody detection *C_*t*_*-values <37	Bio-Germ, Shanghai (sensitivity 96.15% specificity 100.0%)	No clinical symptoms	
Yuan J. et al., 2020	25/172 discharged patients	14 days, 5.23 ±4.13 days	Cloacal swab and nasopharyngeal swab samples RT-PCR *C_*t*_*-value ≤ 40	AM1005; Thermo Fisher Scientific [sensitivity 85.3% (74.6–92.8) Specificity 100% (95.8–100)]	No clinical symptoms	
Zhang B. et al., 2020	7 pts	7–11 days	Throat or rectal swabs RT-PCR	No specified	Asymptomatic	
Zhang J.-F. et al., 2020	1pt	4 days	Throat swab sample RT-PCR	No specified	Asymptomatic	
Zhang R. Z. et al., 2020	4 pts	14–21 days	Nasopharyngeal swab RT-PCR	No specified	Asymptomatic	
Zhao et al., 2020	7/14 pts	7–17 days	Nasopharyngeal swab samples RT-PCR *C_*t*_*-value ≤ 40	Zhongshan Daan Biotech (sensitivity and specificity 100%)	Asymptomatic	
Zheng et al., 2020	3/20 pts after hospital discharge	7 days	Salivary tests RT-PCR and fecal nucleic acid (RNA) test	No specified	No symptoms	
Zhou H. et al., 2020	40-year-old male	5 days after	Sputum or nasopharyngeal swab specimens RT-PCR ORF 1a/1b and nuclear gene *C_*t*_*-values ≤ 40	No specified	Higher density of consolidation on chest CT	
Zhou X. et al., 2020	17/98 pts	3–8.5 days	Oropharyngeal swab RT-PCR. Total exon sequencing	No specified	12 fever, 8 cough, 1 diarrhea	4 fatigue
Zhou Y. et al., 2020	53/257 pts	During quarantine	Throat swabs RT-PCR	No specified	Two patients developed clinical symptoms: one of the patients developed a cough, while the other patient had diarrhea	

*RT-PCR, reverse transcriptase polymerase chain reaction; qRT-PCR, quantitative reverse transcriptase polymerase chain reaction; Ct, cycle threshold; ORF1ab, open reading frame1ab; N, nucleocapsid gene; E, envelope gene; RdRP, RNA-dependent RNA polymerase genes*.

The clinical picture and subsequent ENT are compatible with a diagnosis of the spontaneous acute vestibular syndrome. The most common cause is an acute peripheral vestibulopathy known as vestibular neuritis, affecting the vestibular nerve or “pseudoneuritis” if the acute lesions affect the root entry zone of the eighth nerve or the vestibular nucleus (Wu Y. et al., 2020).

The neuroinvasive potential of SARS-CoV-2 is highlighted by some studies (Baig, 2020; Magnavita et al., 2020).

A relapse of the disease with the involvement of the nervous system may indicate that the virus can be neurotropic since the beginning of the disease or in its recurrence form.

The virus may reach the central nervous system *via* the olfactory nerve. Olfactory and gustatory dysfunctions without rhinorrhea or nasal obstruction are distinctive of patients with mild-to-moderate COVID-19 infection (Baig, 2020; Cooper et al., 2020; Magnavita et al., 2020; Paniz-Mondolfi et al., 2020; Wu Y. et al., 2020), leading to speculation regarding the olfactory nerve as a possible route of the central nervous system entry.

Dizziness is a common onset symptom of COVID-19 (Table 2). This symptom is often considered a non-specific neurological manifestation and is not actively researched or detailed in the description of the clinical picture. This can lead to variability of prevalence estimates, ranging from 3 to 16% between studies. Dizziness such as headache, fatigue, and myalgia are all likely to be caused by the systemic condition if not well-characterized. Specific vestibular or hearing impairment is rarely reported (Table 2). Vertigo should be investigated in SARS-CoV-2 patients and considered along with neurological signs induced by the invasion of the vestibular pathway from the nerve to the vestibular nuclei complex. It is plausible to hypothesize that if the SARS-CoV-2 can also reach the brain from the lungs through the vagus nerve, the virus will invade the brainstem starting with the vagal nucleus and surrounding sites, including the respiratory control center and more, which can lead to more respiratory dysfunction that further exacerbates the damage caused by the primary infection in the lungs or others neurological symptoms (Lukiw et al., 2020; Yachou et al., 2020), such as vestibular impairment. This hypothesis is supported by the evidence of the presence of a consistent angiotensin-converting enzyme (ACE2) expression across the cerebral cortex. The highest ACE2 expression was found in the pons and the medulla oblongata (Guan et al., 2020). Indeed, SARS-CoV-2 appears to bind exclusively to the ACE2 protein, a single-pass type 1 transmembrane receptor with its enzymatically active domain exposed on the surface of multiple cell types, such as type II alveolar cells of the respiratory system, enterocytes and intestinal epithelial cells, endothelial cells, epithelial cells of the conjunctival epithelium, kidney cells (renal tubules), and certain immune cells, such as the alveolar monocytes/macrophages and certain cells of the CNS including those of the cerebral cortex, especially the brainstem (Zubair et al., 2019; Chigr et al., 2020; Kabbani and Olds, 2020; Li C. et al., 2020; Li M. et al., 2020; Panupattanapong and Brooks, 2020; Zhou L. et al., 2020; 154). The highest levels of ACE2-expression in the brain were found in the pons and medulla oblongata, the breathing centers of the brain, which may in part explain the unusually strong ability of SARS-CoV-2 to disrupt normal respiration and pulmonary manifestations including shortness of breath, impaired breathing, and severe respiratory distress. Significant neuroinvasion involving SARS-CoV-2 has been reported from both patients and experimental animals, where the brainstem was heavily infected from apparent spreading *via* a synapse-connected route to the medullary cardiorespiratory centers (Panupattanapong and Brooks, 2020).

**Table 2:** Dizziness as clinical onset symptom reported in literature.

**References**	**Study**	**Dizziness**	**%**	**Hearing or Vestibular impairment**
Chen T. et al., 2020	Retrospective study	Dizziness	7.66%	
Chern et al., 2021	Case report			Bilateral sudden sensorineural hearing loss, bilateral aural fullness, and vertigo
Chirakkal et al., 2020	Case report			Hearing loss and tinnitus
Correia et al., 2020	Systematic review	Dizziness	13.9 %	
Degen et al., 2020	Case report			Asymmetric and bilateral sudden hearing loss and tinnitus
Di Carlo et al., 2020	Systematic review	Dizziness	13.9 %	
Fadakar et al., 2020	Case report			Progressive vertigo in cerebellitis
Fidan, 2020	Case report			Acute otitis media, hearing loss, and tinnitus
Hu Z. et al., 2020	Retrospective study	Dizziness	4%	
Iltaf et al., 2020	Cross-sectional study	Vertigo	3.4%	
Sia, 2020	Case Report	Dizziness		
Karadaş et al., 2020	Prospective clinical study	Dizziness	6.7%	
Karimi-Galougahi et al., 2020	Case series			6 pts with acute-onset hearing loss and/or vertigo
Kilic et al., 2020	Case report			Sudden hearing loss
Klironomos et al., 2020	Retrospective study (one case)			Vestibular neuronitis
Lamounier et al., 2020	Case report			Sudden hearing loss
Liu et al., 2020b	Case report			Vertigo
Lon et al., 2020	Cross-sectional	Dizziness	20%	
Maharaj and Hari, 2020	Case report			Vertigo and tinnitus
Maharaj et al., 2020	Systematic review			100% hearing loss, 10% associated vestibular symptoms
Malayala and Raza, 2020	Case report			Acute vestibular neuritis
Mao et al., 2020	Retrospective study	Dizziness	16.8%	
Mi et al., 2020	Retrospective study	Dizziness	33%	
Özçelik Korkmaz et al., 2020	Case series	Dizziness	31.8%	Tinnitus (11%), true vertigo (6%), hearing impairment (5.1%)
Qin et al., 2020	Case series	Dizziness	8.1%	
Romero-Sánchez et al., 2020	Retrospective study	Dizziness	6.1%	
Shahriarirad et al., 2020	Case series	Dizziness/vertigo	39.8%	
Sriwijitalai and Wiwanitkit, 2020	Case series			Six cases of patients with sudden hearing loss
Sun et al., 2020	Case report			Bilateral hearing loss and tinnitus
Viola et al., 2020	Multicentric study	Dizziness	94.1%	Thirty-four patients (18.4%) reported equilibrium disorders after COVID-19 diagnosis. Of these, 32 patients reported dizziness (94.1%) and 2 (5.9%) reported acute vertigo attacks. Forty-three patients (23.2%) reported tinnitus; 14 (7.6%) reported both tinnitus and equilibrium disorders.
Wang D. et al., 2020	Case series	Dizziness	6.5%	
Zhong et al., 2020	Retrospective study	Dizziness	10.4%	

A limitation of this case consists of the absence of magnetic resonance documentation of vestibular impairment and the genetic characterization of the viruses at the onset and recurrence of COVID-19. The rapid resolution of clinical symptoms within a few days and the trend of not submitting the non-hospitalized patient to neuroimaging exam above all in patients with low suspicions of CNS disease and plan for outpatient ENT visit in the pandemic period, prompted the emergency physician not to proceed. Several causes for repositive tests for SARS-CoV-2 in COVID-19 patients during the recovery period have been described. They include false RT-PCR results or positive due to traces of the RNA genome, intermittent virus shedding, viral reactivation in people with low antibody levels or immunity, reinfection with another SARS-CoV-2 strain, an acute severe systemic inflammatory response known as cytokine release syndrome (CRS), or exposure to a contaminated environmental surface after discharge (Yang et al., 2020; Dao et al., 2021). Various molecular diagnostic assays have been developed and used worldwide, but the differences in their diagnostic performances remain poorly understood (Matsumura et al., 2020; Liotti et al., 2021; Wang M. et al., 2021). Most of the articles do not report the commercial kit used for RT-PCR, and, where reported, the sensitivity and specificity data for the kit is not often available in the literature (Table 1). All the assays exhibited a specificity of 100%, while sensitivity varied (Table 1). The RT-PCR test cannot distinguish between live and dead viruses, but most recurrence of positive RT-PCR is expressed in an asymptomatic way; therefore likely due to dead viruses. We did not perform a genetic characterization of the viruses in order to distinguish between reinfection and reactivation of SARS-CoV-2 in our repositive patient.

## Conclusions

This case is suggestive of colonization of the nervous system that can also result in clinical manifestations in cases of recurrence witnessing the diffusion or permanence of SARS-CoV-2 in the nervous system. It also suggests the neurotrophic hypothesis with the possibility of brainstem invasion (pons and medulla oblongata) and the possibility of recurrence with a SARS-CoV-2 positive RT-PCR test and of clinical recurrence with specific neurological symptoms.

Neurological symptoms should be sought and typified in each SARS-CoV-2 patient.

With the outbreak of COVID-19, to better manage the current phase of the pandemic, we should be vigilant for the presence of any neurological symptoms, both as an onset and as a recurrence of infection.

On the basis of the literature examined and reviewed, for recurrence cases and vestibular symptoms during COVID-19, to our knowledge, this is the first case of recurrence with vestibular impairment as neurological symptom, and we suspect it is likely due to viral reactivation. The PCR retest positivity cannot differentiate between reinfectivity and relapse, and dead-viral RNA detection, serological antibody testing, and viral genome sequencing could be always performed in recurrence cases.

## Data Availability Statement

The raw data supporting the conclusions of this article will be made available by the authors, without undue reservation.

## Ethics Statement

Ethical review and approval was not required for the study on human participants in accordance with the local legislation and institutional requirements. The patients/participants provided their written informed consent to participate in this study. Written informed consent was obtained from the individual(s) for the publication of any potentially identifiable images or data included in this article.

## Author Contributions

All authors equally contributed to the conception and design of the study. All the authors agreed on the previous version of the manuscript, read, and approved the final manuscript.

## Author Disclaimer

As for FG, Medical Director of the Italian Ministry of Health, the opinion and contents expressed in the study are the sole responsibility of the author, and they are not attributable in any way to the institutional and functional positions held by the same at the Italian Ministry of Health (Article 12, paragraph 6, of the Code of Conduct of the Italian Ministry of Health, adopted with DM March 6, 2015 and later).

## Conflict of Interest

The authors declare that the research was conducted in the absence of any commercial or financial relationships that could be construed as a potential conflict of interest.

## Publisher's Note

All claims expressed in this article are solely those of the authors and do not necessarily represent those of their affiliated organizations, or those of the publisher, the editors and the reviewers. Any product that may be evaluated in this article, or claim that may be made by its manufacturer, is not guaranteed or endorsed by the publisher.
